# Pregnancy-induced hypertension awareness, knowledge and its risk factors: A cross-sectional study

**DOI:** 10.12669/pjms.40.4.8247

**Published:** 2024

**Authors:** Bulelwa Beatrice Peter, Uchenna Benedine Okafor

**Affiliations:** 1 Bulelwa Beatrice Peter, Department of Public Health, University of Fort Hare, East London, South Africa; 2 Uchenna Benedine Okafor, Faculty of Health Sciences, University of Fort Hare, East London, South Africa

**Keywords:** Hypertension, Pregnancy-Induced Hypertension, Maternal Mortality, Awareness, Knowledge, Behaviour

## Abstract

**Background and Objective:**

Pregnancy-induced hypertension (PIH) has severe implications for maternal morbidity and mortality; thus, boosting pregnant women’s awareness and knowledge of this medical condition is crucial for improving the mother’s and foetus’s health. This study assessed the awareness and knowledge of PIH and its risk factors among pregnant women in Mdantsane, South Africa.

**Methods:**

This cross-sectional study involved 249 conveniently selected and consenting pregnant women attending antenatal care clinics in Mdantsane, Buffalo City Metropolitan Municipality, South Africa. A self-designed questionnaire was utilised to collect data. Descriptive statistics, chi-square (χ2) test and multivariate logistic regression analysis were performed. The significance level was 0.05.

**Results:**

Over 50% of the women were knowledgeable about PIH and associated risk factors ((χ2=4.92; p = 0.04). The prevalence of PIH was 51.8%, and married women were more aware of the PIH risk factors (71.1%). Women with previous pregnancies were more likely to be aware of PIH (OR = 17.1, 95%; CI = 9.09 to 32.15) compared to first time mothers. Women in age group 36-45 were 2.5 times more likely to be aware of PIH (OR = 2.5, 95% CI: 1.19–3.24) compared to women aged <35 years. Likewise, women aged 36-45 years were two times more likely to be knowledgeable about risk factors for PIH (OR = 2.3, 95% CI: 1.14–2.81) compared to women aged <35 years. Married women were more likely to be aware of PIH risk factors (OR = 2.70, 95% CI = 1.35-5.47) than unmarried women. Moreover, pregnancy increases the likelihood (OR=12.8, 95% CI: 6.97-23.58) of being aware of PIH risk factors. There was a significant difference between the mean ages of women who knew about PIH risk factors and those who do not (t=3.49, Mean difference = 3.49, p=0.0001, 95% CI (2.54; 4.44)).

**Conclusion:**

The prevalence of PIH was high. Age, history of PIH, previous pregnancy, and marital status were predictors of PIH knowledge/awareness and risk factors for PIH. Context-specific health education programmes during prenatal visits are crucial to improving pregnant women’s knowledge of PIH.

## INTRODUCTION

Worldwide, evidence demonstrates that pregnancy-induced hypertension (PIH) is a dangerous medical condition and a direct cause of maternal morbidity and mortality.^1^,^2^ Not only does it contribute to the latter, but is also associated with prolonged chronic hypertension, renal failure, or neurological disorders, which may, in turn, contribute to most pregnancy-related health complications.^3^-^5^ Research has established maternal mortality and morbidity as the worse health issue on a universal scale. Global estimations demonstrate that 287 000 women died from a maternal cause during 2020, which equates to about 800 maternal deaths per day.^6^ Notably, among the direct obstetric variables, hypertensive disorders remain the most prevalent and the primary driver of maternal and perinatal morbidity and mortality worldwide.^7^,^8^ Globally, PIH accounts for approximately 14% of maternal mortality;^8^ in contrast, 10-15% of maternal deaths occur in low-middle -income countries.^7^ The global prevalence of HDP is 116.4/100,000, with Africa having the highest mean prevalence of 334.9 per 100,000 reproductive women.^8^ The primary risk factors for HDP are primiparity, consumption of alcohol, and multiple pregnancy; other underlying risk factors include age, previous HIP history, family history, pre-existing health issues, obesity, poor diet, environmental variables, and socioeconomic status.^8^ Persistent headache, a new start of vision distortion, loss of consciousness, and continuous right upper quadrant discomfort or epigastric pain, vomiting and nausea, the swelling of hands and face, and convulsion are all indicative signs and symptoms of pregnancy-induced hypertension.^9^

Furthermore, research indicates that a concerning number of teenage deaths are linked to PIH in Africa.^1^ Consequently, health education and awareness programmes targeting pregnant women will reduce PIH-related mortality and morbidity; thus, developing initiatives to achieve Sustainable Development Goals (SDGs) 3.1 and 3.2. As a result, it would be possible to ensure early diagnosis of normotensive females with PIH in pregnancy ≥ 20 weeks gestation.

PIH-related illnesses in South Africa are serious medical impediments to pregnancies, with frequencies varying between 70-80%.^10^ Hypertension, another major cause mortality in South Africa, is estimated to be 50.4%,^11^ with a low awareness (45%) and control (16%) rates.^11^ Another study conducted among 7,303 Black South Africans indicated a 44.4% prevalence rate of hypertension, with 71.7%, 83%, and 54.5% being aware, receiving treatment, and controlled, respectively.^12^ Pregnant women constitute a subset of hypertensive patients who are rarely studied. In South Africa, most maternal deaths due to hypertensive disorders occur in girls younger than 20 years, compared to the total estimated population of women.^3^,^5^ This could be attributable to a lack of knowledge or ignorance about the subject among adolescents and young women. Unintended pregnancies are common (41.9%) among adolescent girls and young women in South Africa, with almost all of these (26.3%) ending in abortions;^13^ this may explain the higher prevalence of HIP in this population in South Africa. In addition, a lack of knowledge regarding PIH signs and symptoms may lead to limited health-seeking behaviour. While researchers speculate that this particular knowledge gap among pregnant women may be due to the influence of traditional healers, there is little evidence supporting this. Given that most women are unaware of the above, it is crucial to identify these gaps and how they relate to maternal and perinatal mortality and morbidity. In order to improve the knowledge of the condition and foster a positive attitude among pregnant women, their health professionals are required to highlight any potential PIH-related consequences.

Various adverse health outcomes arise due to a lack of awareness leading to inadequate utilisation of healthcare facilities, poor health status, low numbers of diagnoses, and poor drug adherence.^14^ Global maternal mortality ratio (MMR) estimates for 2020 stood at 223/100,000 live births; however, the MMR in SSA remains unacceptably high at 654/100,000 live births, while in South Africa, it has declined to 127/100,000 live births.^6^ The lack of progress in decreasing maternal mortality across geographical contexts is due to a variety of circumstances, particularly in less-developed and resource-constrained settings. These include postpartum haemorrhage, pre-eclampsia and hypertensive disorders, pregnancy-related infections, complications of unsafe abortion, and infectious and non-communicable diseases; however, other factors relate to health system failures, such as poor quality of health care, a shortage of skilled health workforce and essential medical supplies, poor governance and corruption, as well as socioeconomic and demographic variables.^6^ Within the South African context, the challenge, however, is that young women, especially those in rural areas, are at a higher risk of developing complications of PIH due to factors such as late bookings at antenatal clinics or unbooked pregnancy-related clinic visits related to transport-related issues.^15^ Based on research conducted in South Africa, young pregnant women are often abused and humiliated by nursing staff and, as a result, are often reluctant to visit health facilities during early pregnancy, which leads to problems.^16^ The resultant effect of this frustration at antenatal clinics and hospitals caused by some ill-mannered healthcare workers is the preference for pregnant women to be cared for by their relatives.^17^

From a clinical perspective, hypertensive disorders of pregnancy (HDP) have been found to have negative effects on both the mother and the foetus, leading to various unfavourable perinatal outcomes. These adverse effects include respiratory distress syndrome,^18^,^19^ congenital deformities in newborns,^20^ stillbirth and neonatal death,^18^ as well as preterm birth, placental abruption, and postpartum haemorrhage.^19^ Consequently, it is crucial to implement interventions that centre on prenatal assessments, health awareness programmes, and modifiable risk factors. Improving pregnant women’s awareness about risk factors of PIH may be beneficial. In addition, it is important to monitor and control PIH in pregnant women to avoid detrimental consequences on both the mother and the foetus. Although, many published PIH studies exist in other settings, such information is lacking in Mdantsane, a resource-scarce semi-urban large black township settlement in the Eastern Cape of South Africa. Pregnant women’s awareness, behaviour towards PIH and its risk factors would inform public health policy adjustment to improve maternal health outcomes. Decision-making is shaped by information. Such knowledge would assist in improving HIP interventions in the setting. Therefore, this study assessed the awareness, knowledge and risk factors of HIP among pregnant women attending antenatal health facilities in Mdantsane of the Eastern Cape Province, South Africa.

## METHODS

This cross-sectional study was conducted at eight PHCs in Mdantsane in South Africa’s Eastern Cape Province. Mdantsane is the country’s second-largest township with eighteen zones and the newest developed unit P. Mdantsane has a very high unemployment rate with a population of 250,000. Most residents live below the breadline, according to the nation’s economic classifications. There are eight primary health centres (PHC), one community health centre (Nontyatyambo CHC), and one regional hospital (Cecilia Makhiwane Hospital). The CHC and the hospital are referral centres in cases requiring further management.

### Design, population and sampling

This cross-sectional study was conducted on 249 pregnant women attending antenatal healthcare clinics at eight PHCs in Mdantsane at Buffalo City Municipality, East London. Convenient sampling was used to select both the clinics and the participants. Inclusion criteria were pregnant women who had attended antenatal care at the eight PHCs.

### Data collection

All pregnant women were approached and those who agreed to participate in the study received an informed consent form. The study’s objectives and procedures were thoroughly explained to the participants. During data collection, confidentiality and privacy were maintained since the data in the completed questionnaire was anonymized. Data was collected by utilising a self-administered questionnaire containing closed-ended questions.

### Measures

Primary outcome measure: Pregnancy-induced hypertension (knowledge and awareness of the risk factors of PIH).

Literature pertinent to the research objectives served as the basis for a self-designed questionnaire.^21^-^23^ The participants’ knowledge about PIH signs and symptoms, risk factors, prevention, complications, and management were assessed through the use of questionnaire. The questionnaire comprised two types of questions: multiple-choice and closed-ended, with responses (’yes, no, don’t know’). Similarly, awareness centred on the definition of PIH, its prevention, its signs and symptoms, and its complications and management. Knowledge and awareness scored one and zero points for correct and wrong answers, respectively. The sum of correct responses was computed. A cut-off of >6 indicates good knowledge and awareness, whereas a cut-off of six indicates poor knowledge and awareness, respectively.

### Covariates

Participants’ socio-demographic variables assessed were age, religion, marital status, educational level, and employment status. In addition, medical variables assessed include: previous history of pregnancy, previous delivery, and use contraceptive usage.

### Ethical considerations

The University of Fort Hare’s Ethics Review Committee approved the study protocol (GOO031SPET01). Prior to data collection permission obtained from the Eastern Cape Provincial Department of Health, and from health managers of the selected health facilities for the study. Written informed consent was obtain before data collection. The research was conducted in accordance with the Helsinki Declaration’s ethical research principles.

### Data analysis

Descriptive statistics were applied to report the frequency counts, percentages, means and standard deviation (SD). In addition, two independent t-test samples examine was applied to examine the effect of age on participants’ awareness and knowledge of PIH and its risk factors. Furthermore, the chi-square was utilised to examine comparisons between groups. In addition, the multivariate logistic regression analysis was performed to the factors related to HIP among the pregnant women. All statistical analyses were performed using the Statistical Package for Social Sciences (SPSS) version 29.0. A 0.05 p-value indicated statistical significance.

## RESULTS

Population summarizes in Table-I by the socio-demographic characteristics. The majority of participants were aged 26-35 (45.0%), single women (82%), unemployed (74.7%), and most of whom having only completed secondary education (n=123, 49.4%) (Table-I).

**Table-I:** Socio demographic characteristics of the study population.

Variables	n (%)
***Age (years)***	
19-25	71 (28.5)
26-35	112 (45.0)
36-45	66 (26.5)
***Religion***	
Anglican	88 (35.3)
Apostolic	18 (7.2)
Baptist	2 (0.8)
Jehovah’s Witness	13 (5.2)
Jesus Christ Church	6 (2.4)
Methodist	61 (24.5)
Presbyterian	33 (13.3)
Seventh Day Adventist	6 (2.4)
Zion	22 (8.8)
***Marital status***	
Married	45 (18.1)
Single	204 (81.9)
***Educational level***	
Secondary	123 (49.4)
Diploma	90 (36.1)
University	36 (14.5)
***Employment status***	
Unemployed	186 (74.7)
Employed	63 (25.3)
***Previous pregnancy***	
Yes	140 (56.2)
No	109 (43.8)
***Previous delivery***	
Yes	140 (56.2)
No	109 (43.8)
***Contraceptive usage***	
None	48 (19.3
Condom	102 (41.0)
Injectable	78 (31.3)
Pill	21 (8.4)

Pregnancy and PIH knowledge are shown in Table-II. Over 50% of participants knew about PIH and its risk factors (χ2=4.92; p=0.04), whereas 43.8% of women did not. About 56.2% had given birth before, while 51.8% had PIH, and 57.0% were aware of it. Compared to unmarried women, 71.1% of married women knew of PIH risks.

**Table-II:** Pregnancy history and knowledge of PIH.

Variable	Response [n (%)]		χ2	p-value

	Yes	No		
Previous pregnancy	140 (56.2)	109 (43.8)	3.86	0.05
Previous delivery	140 (56.2)	109 (43.8)	3.86	0.05
Awareness of PIH	142 (57.0)	107 (43.0)	4.92	0.04^s^
Knowledge of PIH risk factors	129 (51.8)	120 (48.2)	0.33	0.57

S: statistically significant.

The findings indicated that women in age group 36-45 were 2.5 times more likely to be aware of PIH (OR = 2.5, 95% CI: 1.19–3.24) compared to women aged <35 years. Also, married women were more likely to be aware of PIH than single women (OR=2.4, 95% CI; 1.17-4.90) while, compared to women having their first pregnancy, those with previous pregnancies were more likely to be aware of PIH (OR=17.1, 95% CI; 9.09-32.15). No statistically significant association was found between the educational level of the respondents and their awareness of PIH (Table-III).

**Table-III:** The association between participants’ socio demographic characteristics and awareness of PIH.

Characteristic		Total n (%)	PIH Awareness n (%)		OR	95% CI

			Aware	Not Aware		
Age	19-25	71 (28.5)	41 (57.7)	30 (42.3)	Reference	
	26-35	112 (45.0)	65 (58.0)	47 (42.0)	0.8	(0.6-1.23)
	36-45	66 (26.5)	36 (54.5)	30 (45.5)	2.5	(1.19-3.24)^s^
Marital status	Married	45 (18.1)	33 (73.3)	12 (26.7)	2.4	(1.17-4.90)^s^
	Single	204 (81.9)	109 (53.4)	95 (46.6)	Reference	
Education	High School	123 (49.4)	65 (52.8)	58 (47.2)	0.9	(0.63-1.27)
	Diploma	90 (36.1)	51 (56.7)	39 (43.3)	0.8	(0.50-1.16)
	Degree	36 (14.5)	26 (72.2)	10 (27.8)	Reference	
Previous pregnancy	Yes	140 (56.2)	117 (83.6)	23 (16.4)	17.1	(9.09-3.15)^s^
	No	109 (43.8)	25 (22.9)	84 (77.1)	Reference	

Participants’ knowledge of PIH-related risk factors also highlighted (Table-IV). Women aged 36-45 years were two times more likely to be knowledgeable about risk factors for PIH (OR = 2.3, 95% CI: 1.14–2.81) compared to women aged <35 years. Married women were more likely than single women to know the risk factors of PIH (OR=2.7, 95% CI; 1.35-5.47). Furthermore, the findings indicated that being pregnant increased women’s chances (OR=12.8, 95% CI; 6.97-23.58) of awareness about the related risk factors.

**Table-IV:** The association between socio demographic characteristics and knowledge of PIH risk factor.

Characteristic		Total n (%)	PIH risk factor knowledge		OR	95% CI

			Know	Don’t Know		
Age	19-25	71 (28.5)	37 (52.1)	34 (47.9)	Reference	
	26-35	112 (45.0)	58 (51.8)	54 (48.2)	0.7	(0.5-1.73)
	36-45	66 (26.5)	34 (51.5)	32 (48.5)	2.3	(1.14-2.81)^s^
Marital status	Married	45 (18.1)	32 (71.1)	13 (28.9)	2.7	(1.35-5.47)^s^
	Single	204 (81.9)	97 (47.5)	107 (52.5)	Reference	
Education	High School	123 (49.4)	56 (45.5)	67 (54.5)	1.2	(0.84-1.71)
	Diploma	90 (36.1)	49 (54.4)	41 (45.6)	0.8	(0.55-1.27)
	Degree	36 (14.5)	24 (66.7)	12 (33.3)	Reference	
Previous pregnancy	Yes	140 (56.2)	107 (76.4)	33 (23.6)	12.8	(6.97-23.58)^s^
	No	109 (43.8)	22 (20.2)	87 (79.8)	Reference	
Contraceptive used	None	48 (19.3)	22 (45.8)	26 (54.2)	1.2	(0.67-2.08)
	Condom	102 (41.0)	53 (52.0)	49 (48.0)	0.9	(0.63-1.36)
	injectable	78 (31.3)	37 (47.4)	41 (52.6)	1.1	(0.71-1.73)
	Pill	21 (8.4)	17 (81.0)	4 (19.0)	Reference	

S: statistically significant.

Two independent t-test sample was used to determine the effect of age on participants’ awareness and knowledge of PIH and its risk factors (Table-V). There was a statistically significant difference between the mean age of those who knew about the condition (t=6.97, Mean difference = 3.45, p<0.0001, 95% CI; 2.47-4.42) and its risk factors (t=3.49, Mean difference = 3.49, p<0.0001, 95% CI; 2.54-4.44), and those who did not.

**Table-V:** Age and PIH awareness and knowledge of PIH risk factors.

Characteristic		N	Mean	SD	t	p-value	Mean Difference	95% CI
PIH awareness	Aware	142	25.1	4.443	6.97	<0.0001	3.45	(2.47-4.42)^s^
	Not Aware	107	21.6	2.918				
PIH risk factor knowledge	Know	129	25.2	4.551	3.49	<0.0001	3.49	(2.54-4.44)^s^
	Don’t Know	120	21.8	2.901				

S: statistically significant.

## DISCUSSION

Our finding demonstrated that half of the participants knew about PIH and its risk factors. Women with a history of PIH were more likely to be aware of the risk factors of HIP than their peers with a history of PIH. In addition, women with previous pregnancies were more likely to be aware of the risk factors for PIH. We also found that age was associated with PIH knowledge and risk factors in our sample of women. Furthermore, married women were more likely to be aware of the risk factors for PIH than their single counterparts. These results highlight the need for context-specific health interventions to improve antenatal care as well as early diagnosis and management of pregnancy-induced hypertension in this understudied, poor, semi-urban population.

In this study, half of the participants were aware of PIH and its risk factors. Previous research has found comparable results.^24^-^26^ Previous studies undertaken in South Africa^22^ and elsewhere^27^-^29^ have found that patients had inadequate understanding regarding PIH, complications, and risk factors. Notably, some of these studies attributed the lack of awareness and knowledge about PIH to misconceptions and myths, such as women believing that PIH is a spiritual condition that requires spiritual treatment^27^,^30^,^31^ or a sign that a woman is likely to birth a baby boy.^32^ Inadequate awareness of PIH may exacerbate the maternal health burden associated with PIH. Furthermore, incorrect perceptions regarding PIH may impede early health seeking, leading to late diagnosis and treatment, which could have negative health consequences for the mother and baby. However, our study did not assess cultural misrepresentations of PIH; however, our findings highlight the importance of providing women with proper health education and counselling about PIH during antenatal care. A prior investigation has indicated that the provision of an antenatal treatment package to women during their prenatal visits has been shown to be useful in enhancing their understanding of pregnancy-induced hypertension.^33^ Therefore, it is advisable that women adhere to regular clinic check-ups during pregnancy to ensure they are empowered to improve their behaviour in this regard and maintain blood pressure control.^34^

The current study found that women with a history of PIH were more likely to be aware of the risk factors of HIP than their peers with a history of PIH. Previous research has found that a history of previous PIH is an independent risk factor for the development of PIH.^35^-^37^ A similar finding was found in Meazaw et al.^38^ review study in SSA. This particular finding suggests that women having a history of past PIH should be prioritised and checked for PIH. A family history of PIH suggests genetic and shared environmental or behavioural variables that may contribute to PIH risk; and family history of hypertension is a significant and easily accessible HDP risk factor.^39^

Women with previous pregnancies were more likely to be aware of the risk factors for PIH than those who were pregnant for the first time, which is consistent with earlier findings in India,^29^ Iraq,^21^ and South Africa.^22^ Other studies conducted in Ghana^28^ and India^35^ found contrasting findings. It is probable that women with high parity are more inclined to attend regular prenatal visits and are well-informed and aware about PIH, possibly due to their participation in counselling or educational programmes on the subject. Unlike their nulliparous peers, who may have no reason to attend antenatal clinic. Higher levels of awareness can be achieved through the health education of the at-risk population. Consequently, this will reduce PIH-related mortality and morbidity and contribute to achieving MDGs. As established, PIH mortality remains high due to expectant women’s ignorance, with some attributing the disease’s symptoms to witchcraft rather than their physical causes.^5^ This necessitates a targeted strategy and approach on the part of the Department of Health to ensure that pregnant women receive this vital information during prenatal visits.

In addition, younger pregnant women (25 years or younger) are more likely to be uneducated and unaware of PIH than their older counterparts. Consequently, maternal age is an important factor related to PIH; furthermore, since the older the woman, the higher her odds of developing it. This is consistent with findings from Thailand,^40^ Bangladesh^41^, and Ethiopia.^42^ Other researchers, however, reported no significant association between PIH knowledge/risk factors and participant’s age.^43^ Teenagers are more likely to have a risk factor for PIH during pregnancy.^39^ Notably, teenagers and young women use oral contraceptives to avoid pregnancy, and there is evidence that this contributes to the development of PIH.^44^ A recent study found that teenage births are more likely to result in eclampsia-related maternal fatalities.^45^ As such, teenage blood pressure should be a priority in prenatal care.

Consistent with prior studies,^23^,^39^,^46^,^47^ married women were more likely than single women to be knowledgeable of PIH risk factors. Other studies, however, found conflicting results.^47^-^49^ The substantial relationship between marital status and risk factor for PIH can most likely be explained by either of two possibilities. First, there is the likelihood of minimal preconception seminal fluid interaction between women and their partners, as well as the stress of giving birth without spousal or family social and economic support.^39^

In contrast to earlier reports, our findings showed that educational level had no association with PIH knowledge.^23^,^33^,^41^,^42^ Other studies, like ours, revealed no significant association between respondents’ levels of knowledge and their educational status.^38^,^43^ Women with a higher education, in contrast, could have better access to information, education, and communication from various sources such as the internet, books, and television, and hence may have more knowledge of PIH than women with little or no education. Kanyamura et al.^50^ echoes that circumstances contributing to low knowledge levels may differ for different individuals. Patients’ understanding of PIH is the most essential factor in ensuring they are positive about how to monitor and control their blood pressure. For an individual to be familiar with this condition, they must first understand and acknowledge it. After receiving the appropriate education/information, most individuals clearly understand the condition; this, in turn, will encourage them to be more positive. In light of this, pregnant women in Mdantsane can only understand PIH-related complications and knowledge of risk factors if they are properly educated about it. According to Fadare,^51^ the higher a woman’s level of education, the more likely she is to acquire knowledge of the condition, cultivate a positive attitude, and change her behaviour in regard to gestational hypertension. In addition, an earlier Nigerian study noted that both healthcare providers and pregnant women were ignorant of the importance of regularly attending an antenatal clinic; this health-associated “illiteracy” contributes to the low levels of women that utilise available antenatal services.^52^

### Limitations of the study

Since the questionnaire was self-designed, instances of untruthfulness and recall bias cannot be disregarded. Furthermore, this was a cross-sectional study; therefore, it did not aim to determine further possible causal associations. Finally, additional factors including gravidity and parity were not evaluated. Hence, future studies should endeavour to investigate the interplay between these obstetric risk factors and knowledge, awareness, and risk factors associated with PIH.

## CONCLUSION

In this setting, the prevalence of PIH was high. Age, history of PIH, previous pregnancy, and marital status were predictors of PIH awareness, knowledge and risk factors for PIH. There is a need for community education regarding the risk factors of chronic hypertension and the significance of early and regular antenatal clinic visits by pregnant women.

### Author`s Contribution:

**BBP:** Conceptualized the study, collected data.

**UBO:** Drafted the manuscript, Read, and critically reviewed the manuscript.

All authors contributed to the writing of the manuscript, read and approved the final version of the manuscript.
